# Inference-assisted intelligent crystallography based on preliminary data

**DOI:** 10.1038/s41598-019-48362-3

**Published:** 2019-08-22

**Authors:** Manabu Hoshino, Yoshinori Nakanishi-Ohno, Daisuke Hashizume

**Affiliations:** 1 PRESTO, Japan Science and Technology Agency (JST), 4-1-8 Honcho, Kawaguchi, Saitama 332-0012 Japan; 2 RIKEN Center for Emergent Matter Science (CEMS), 2-1 Hirosawa, Wako, Saitama 351-0198 Japan; 3 Graduate School of Arts and Sciences, The University of Tokyo, 3-8-1 Komaba, Meguro, Tokyo 153-8902 Japan; 4 Komaba Institute for Science, The University of Tokyo, 3-8-1 Komaba, Meguro, Tokyo 153-8902 Japan

**Keywords:** X-ray diffraction, Chemical physics

## Abstract

Crystal structure analysis is routinely used to determine atomically resolved molecular structures and structure-property relationships. The accumulation of reliable structural characteristics obtained by crystal structure analysis has forged a robust basis that is frequently used in molecular and materials sciences. However, experimental techniques remain hampered by time-consuming ‘blind’ measurement-analysis iterations, which are sometimes required to find appropriate crystals and experimental conditions. Herein, we present a method that uses a small preliminary data set to evaluate the to-be-observed structures and the to-be-collected data. Moreover, we demonstrate the practical utility of this method to improve the efficiency of crystal structure analysis. This method will help selecting suitable crystals and choosing favorable experimental conditions to generate results that satisfy the level of precision required for specific research objectives.

## Introduction

The molecular structure of a given compound in the solid state is of particular interest in the context of biological and chemical transformations as well as materials science. Structural observations of molecules and their aggregates promote the interpretation of chemical properties and functions in the context of chemical reactions^1^, phase transitions^2^, morphological change^3^, and energy conversion for mechanical motion^4^. These observations are usually obtained from crystal structure analysis, which has become an indispensable technique in a variety of research areas as it provides the atomically resolved three-dimensional molecular structure in the crystal. Recent developments regarding synchrotrons and free-electron lasers (X-rays) as well as particle accelerators (neutrons) have increased the intensity of these quantum beams for diffraction experiments and led to the long-awaited determination of the structures of proteins^5,6^ and other materials^7,8^. In this context, the development of advanced computer programs should also be noted. The execution of crystal structure analysis has also been facilitated by the development of multifunctional programs that are available for structure analysis^9,10^ and the graphical user interfaces that support the execution of the programs^11,12^.

Yet, when high quality data is required for specific purposes, chemists are sometimes frustrated with the results of crystal structure analysis due to the insufficient geometrical precision, unsolvable structural disorder, or problems^13^ associated with structure evaluation^14^. In spite of the fact that recent packaged software for diffractometer automatically indicates optimized measurement condition for performing crystal structure analysis more easily, selection of a suitable crystal for data collection and decision of favorable X-ray exposure time for precise crystallographic observation (e.g. electron density analysis) are still relied on user’s experience. To obtain robust, precise, and reliable information on molecular structures derived from high quality data, diffraction experiments and the subsequent crystal structure analysis have to be repeated using several crystals and data collection conditions, which is both laborious and time-consuming. One reason that necessitates this repetition of measurements is the lack of diffraction measurements that use a feedback from the to-be-observed molecular structure. Usually, single crystals for measurements and the data-collection time are selected based on a small set of preliminary diffraction data. However, due to the phase problem^15^, diffraction intensity does not directly correspond to the molecular structure, i.e., the phase of a structure factor for identifying the molecular structure is not contained in the diffraction intensity. Only after collection of large data sets, it is possible to retrieve the phases using computational methods such as direct methods^16,17^ or dual-space methods^10,18^. Alas, this information cannot be obtained from the small preliminary data sets. Therefore, the chosen crystal and data-collection time are not guaranteed to provide a satisfactory molecular structure but simply represent a favorable ratio between diffraction intensity and measurement errors.

Herein, we report an efficient crystallographic experimental method for finding a suitable crystal and collecting high quality diffraction data much easier with the aid of the estimation of a parameter in the subject crystal using only a small set of preliminary diffraction data. This method is based on the following equation from Wilson’s statistics^19^:

1 $$\begin{array}{c}\langle I\rangle ={(1/C)}^{2}\times \sum _{j}\,{{f}_{j}}^{2}\exp \{\,-\,2\langle B\rangle {(\sin \theta /\lambda )}^{2}\},\end{array}$$

wherein 〈*I*〉 refers to averaged diffraction intensities (*I*s) on a selected range, *C* to a scaling factor to obtain the absolute *I*, *f*_*j*_ to the atomic scattering factor of the *j*th atom in a unit cell, 〈*B*〉 to an averaged isotropic thermal parameter of atoms in a unit cell, and sin*θ*/*λ* to a resolution given by the diffraction angle (*θ*) and the wavelength of the X-ray or neutron beam (*λ*). This equation bypasses the phase problem and directly provides 〈*B*〉 in a subject crystal from the diffraction data without a crystal structure analysis. The value of 〈*B*〉 thus reflects i) the degree of overall atomic thermal motion, ii) the rigidity, which corresponds to the level of packing, and iii) the regularity, which corresponds to the degree and periodicity of the molecular alignment in the crystalline sample. In the present study, values of 〈*B*〉 were estimated from a small set of preliminary diffraction data and used to evaluate the to-be-observed structure prior to collecting a full set of diffraction data and carrying out the corresponding crystal structure analysis.

The distribution of the diffraction data also depends on 〈*B*〉. Here, the probability for the collection of *I* at a given resolution was considered. The probability distribution function of *I*, *P*(*I*), is described by

2 $$\begin{array}{c}P(I)=\{\begin{array}{c}{(\Sigma )}^{-1}\exp (-I/\Sigma )\,({\rm{non}} \mbox{-} {\rm{centrosymmetric}})\\ {(2\pi \Sigma I)}^{-1/2}\exp (-I/2\Sigma )\,\,\,\,({\rm{centrosymmetric}}),\end{array}\end{array}$$

whereby

3 $$\begin{array}{c}\Sigma =\sum _{j}\,{{f}_{j}}^{2}\exp \{\,-\,2\langle B\rangle {(\sin \theta /\lambda )}^{2}\}.\end{array}$$

The parameter *Σ* refers to the mean value of the distribution of Eq. (2) for both symmetry cases^20^. Given its statistical nature, intensities lying on a narrow range of sin*θ*/*λ* (a resolution shell) should stochastically follow *P*(*I*). When the number of intensities becomes sufficiently large, 〈*I*〉 approximately corresponds to *Σ*^21^ and Eq. (3) becomes equivalent to Eq. (1) on a relative scale. Then, *P*(*I*) represents a distribution of diffraction intensities in the shell at a given resolution. In the present study, *P*(*I*) was generated from the estimated value of 〈*B*〉, which was obtained from a preliminary small set of diffraction data that was used for the evaluation of the quality of the to-be-collected diffraction data of a specific crystal.

One potential problem for the use of 〈*B*〉 as an evaluation parameter for specific crystals is its inherently low accuracy, which is conventionally estimated using Eq. (1)^19^, i.e., the least-squares fitting of the values of the logarithm of 〈*I*〉 divided by the sum of *f*_*j*_^2^ to the linear function of sin*θ*/*λ*. Even though the accuracy of the thus estimated values of 〈*B*〉 is somewhat diminished due to random errors in 〈*I*〉, this becomes negligible upon increasing the number of 〈*I*〉 for the least-squares fitting. The number of 〈*I*〉s is usually limited as it depends on the selected range of a resolution shell for averaging *I*. Even when a narrow shell [e.g. Δ(sin*θ*/*λ*) = 0.01 Å^−1^] is applied, the number of 〈*I*〉s is less than 100 due to the limit of sin*θ*. This limit decreases even further due to the difficulties associated with the detection of innately weak diffraction intensities in high *θ* regions^22^. Therefore, even when a large data set is collected for crystal structure analysis, a moderately yet sufficiently accurate value of 〈*B*〉 can be estimated using the low number of 〈*I*〉s and later be refined to reach the true value in the subsequent crystal structure analysis.

To estimate highly accurate values of 〈*B*〉 prior to the collection of the full diffraction data set and the crystal structure analysis, Bayesian inference^23^ was used in the present method. Bayesian inference allows the statistical estimation of intrinsic parameters in data sets and is currently used in some crystallographic computer programs for e.g. absolute structure determination^24^ and the correction of negative intensities^25^. As described in Eq. (3), 〈*B*〉 intrinsically belongs to *Σ*, which is observable as 〈*I*〉. In Bayesian inference, the observed *Σ*s at all resolution shells are simultaneously used for updating the prior knowledge of 〈*B*〉 to the unique 〈*B*〉 as the intrinsic parameter of the subject. Here, *S* is defined by Eq. (4), which is used thereafter instead of *Σ* in order to simplify the present description:

4 $$\begin{array}{rcl}S & \equiv  & \Sigma /\sum _{j}\,{{f}_{j}}^{2}\\  & = & \exp \{\,-\,2\langle B\rangle {(\sin \theta /\lambda )}^{2}\}.\end{array}$$

Bayesian inference regards ‘prior knowledge’ and ‘observation of data’ as probability distributions and the aforementioned knowledge update is expressed by Bayes’ theorem:

5 $$\begin{array}{c}P(\langle B\rangle |{\boldsymbol{S}})\propto \,P({\boldsymbol{S}}|\langle B\rangle )P(\langle B\rangle ).\end{array}$$

Here, *P*(〈*B*〉|***S***) and *P*(〈*B*〉) refer to the posterior and prior distribution of 〈*B*〉, while ***S*** refers to the observed data set (*S*_1_, *S*_2_, *S*_3_, ··· *S*_*n*_ from *Σ* and *f*_*j*_s at each resolution shell; *n*: number of shells). The probability of observing ***S*** underlying 〈*B*〉 [*P*(***S***|〈*B*〉), i.e., the likelihood] is derived from Eq. (4) by regarding it as a frequency distribution of the event that occurs *S* times at a given resolution. The probability distribution function of this event, i.e., observing *S* at a given resolution, is derived from normalizing the right side of Eq. (4) (*cf*. Supplementary Note). The likelihood for each *S* [*P*(*S*|〈*B*〉)] is a probability distribution function, which describes that the above event simultaneously occurs *S* times; *P*(***S***|〈*B*〉) in Eq. (5) is provided as the joint probability distribution function of all *P*(*S*|〈*B*〉):

6 $$\begin{array}{rcl}P({\boldsymbol{S}}|\langle B\rangle ) & = & \mathop{\prod }\limits_{m=1}^{n}P({S}_{m}|\langle B\rangle )\\  & \propto  & \mathop{\prod }\limits_{m=1}^{n}{[{(\frac{2\langle B\rangle }{\pi })}^{\frac{1}{2}}\exp \{-2\langle B\rangle {(\sin {\theta }_{m}/\lambda )}^{2}\}]}^{{S}_{m}}\\  & = & {(\frac{2\langle B\rangle }{\pi })}^{\mathop{\sum }\limits_{m=1}^{n}\frac{{S}_{m}}{2}}\exp \{-2\langle B\rangle \mathop{\sum }\limits_{m=1}^{n}{S}_{m}{(\sin {\theta }_{m}/\lambda )}^{2}\}.\end{array}$$

This likelihood represents the update *P*(〈*B*〉) ‘sum of *S*’ times in Eq. (5). In contrast to the small number of 〈*I*〉s for the conventional method using Eq. (1), this update frequency is expected to be sufficiently high to accurately estimate 〈*B*〉, even when only a small set of preliminary diffraction data is collected. In the present method, 〈*B*〉 of a subject is estimated from a small set of preliminary diffraction data by calculation of *P*(〈*B*〉|***S***) using Eqs (4–6) (the implementation of this method is described in detail in the Supplementary Text).

## Results and Discussion

### Reliability of the method

**Figure 1:**
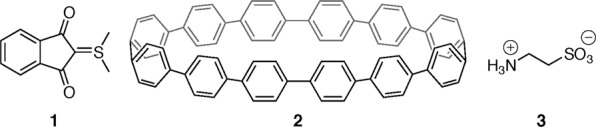
Chemical structures of **1–3**: 2-dimethylsufuranylidene-1,3-indanedione (**1**), [12]cycloparaphenylene ([12]CPP, **2**), and 2-aminoethanesulfonic acid (taurine, **3**). Taurine was found as a zwitterion in the crystal.

One of the standard crystals in chemical crystallography, 2-dimethylsufuranylidene-1,3-indanedione (**1**; Fig. 1), was selected as a sample to evaluate the reliability of the values of 〈*B*〉 estimated using the present method. For that purpose, a preliminary X-ray diffraction data set was collected within one minute (15 frames were collected at 1 s/frame) and used as the subject data set. This data set contained 202 diffraction data points up to the resolution of sin*θ*/*λ* = 0.65 Å^−1^, which is 13.7% of the expected independent diffraction data in this resolution range (Supplementary Data 1). Retrieval of the phases by direct methods (*SHELXS*^16^) or dual-space methods (*SHELXT*^10^) using the subject data set failed due to a shortage of data. A diffraction data set for the crystal structure analysis was subsequently collected maintaining the sample crystal on the diffractometer, and this data set was used as the reference data set that contains 4015 diffraction data points in the same resolution range as the subject data set and a coverage of 99.9%. A crystal structure analysis using the reference data set provided 〈*B*〉 = 1.82(46) Å^2^ (the observed structure of **1** is shown in Supplementary Fig. 1). Using the conventional method and the subject and reference data sets afforded estimated 〈*B*〉 values of 0.88 and 0.93 Å^2^, respectively. These estimated values are beyond the 2σ range of 〈*B*〉 obtained from a crystal structure analysis, which means that the probability for identifying the estimated 〈*B*〉 values by the conventional method from crystal structure analysis is < 5%. The two aforementioned data sets were also used for estimating 〈*B*〉 by Bayesian inference. The prior probability in Bayesian inference can be selected arbitrarily, i.e., prior knowledge is arbitrary expressible therein. In the present study, a uniform distribution was attributed to *P*(〈*B*〉) in Eq. (5) in order to reflect that there is no prior information about 〈*B*〉, i.e., obtaining any value of 〈*B*〉 is equally possible (*cf*. Supplementary Note). In the calculation of the posterior distributions, the *S*s in the shells where no intensity was collected in the subject data set were excluded from the reference data set to compare the estimated value from the same shells. This exclusion changed the number of data points and the coverage in the reference data set to 3579 and 90.6%, respectively. 〈*B*〉 values of 1.64 Å^2^ and 1.62 Å^2^ were estimated from *P*(〈*B*〉|***S***) in Eq. (5) using the subject and excluded reference data set, respectively (all *S*s are summarized in Supplementary Table 1). These values agree well with the result of crystal structure analysis within the range of standard uncertainty. This agreement of 〈*B*〉 demonstrates the ability of the present method to provide reliable 〈*B*〉 values for a given subject crystal.

It should be noted that the absence of collectable *S* affects the estimated value of 〈*B*〉 in the present method. Prior to the aforementioned exclusion of *S*s, the reference data set provided 110 resolution shells upon using 0.005 Å^−1^ for the shell width. Bayesian inference using the reference data set without exclusion provided 〈*B*〉 = 1.86 Å^2^, which corresponds reasonably well with the result of the crystal structure analysis. On the other hand, in the subject data set, 25 out of 110 shells were unavailable due to a lack of coverage in the scanned area and/or X-ray exposure time insufficient to detect weak diffraction intensities during the data collection period (1 min). The effect of unavailable data on the estimated value originates from differences with respect to the resolution dependence of the scattering factor between core and valence electrons^26^. Unavailable *S*s in the subject data set were expected to be collected at low-resolution shells and decreased 〈*B*〉 by lowering the contribution of broadly distributed valence electrons, which is expressed in increments of 〈*B*〉, relative to the diffraction data set (for a detailed description, see the Supplementary Note). This feature of the present method should be kept in mind when comparing isomorphic crystals based on 〈*B*〉 (e.g., evaluation of protein crystals or host-guest exchanges). In such cases, making the effort to collect *S*s in the same shells is strongly recommended if possible to ensure a uniform contribution from core and valence electrons to the data set for the estimation of 〈*B*〉.

### Prior evaluation of molecular structures in isomorphic crystals

Defining a molecular structure in a crystal from the estimation of 〈*B*〉 is in principle impossible given that the characteristics of individual atoms are averaged in this parameter. However, due to the specific features of isomorphic crystals, an evaluation of to-be-observed structure becomes possible by only using the estimated 〈*B*〉 before the crystal structure analysis. As a practical example of the utility of the evaluation of crystal structures based on 〈*B*〉, the evaluation of the crystallinity, which originates from the rigidity and regularity in the crystal packing, of a well-diffracting crystal of a protein has been proposed^27^. In that study, an evaluation of the packing features resulted in a difference of 〈*B*〉 as the crystal specimens are isomorphic, which leads to unique thermal characteristics in the same molecules. The difference of crystallinity between isomorphic protein crystals is sufficiently high to allow a distinction via the moderately accurate conventional estimation of 〈*B*〉. On the other hand, isomorphic crystals of small molecules usually exhibit similar crystallinity and marginal structural differences, which are expected to be reflected in small variations of 〈*B*〉. In the present study, we went beyond the aforementioned crystallinity evaluation of a protein, and used the accurate estimation of 〈*B*〉 obtained from Bayesian inference for the distinction of to-be-observed structures of small molecules in isomorphic crystals prior to their crystal structure analysis.

**Figure 2:**
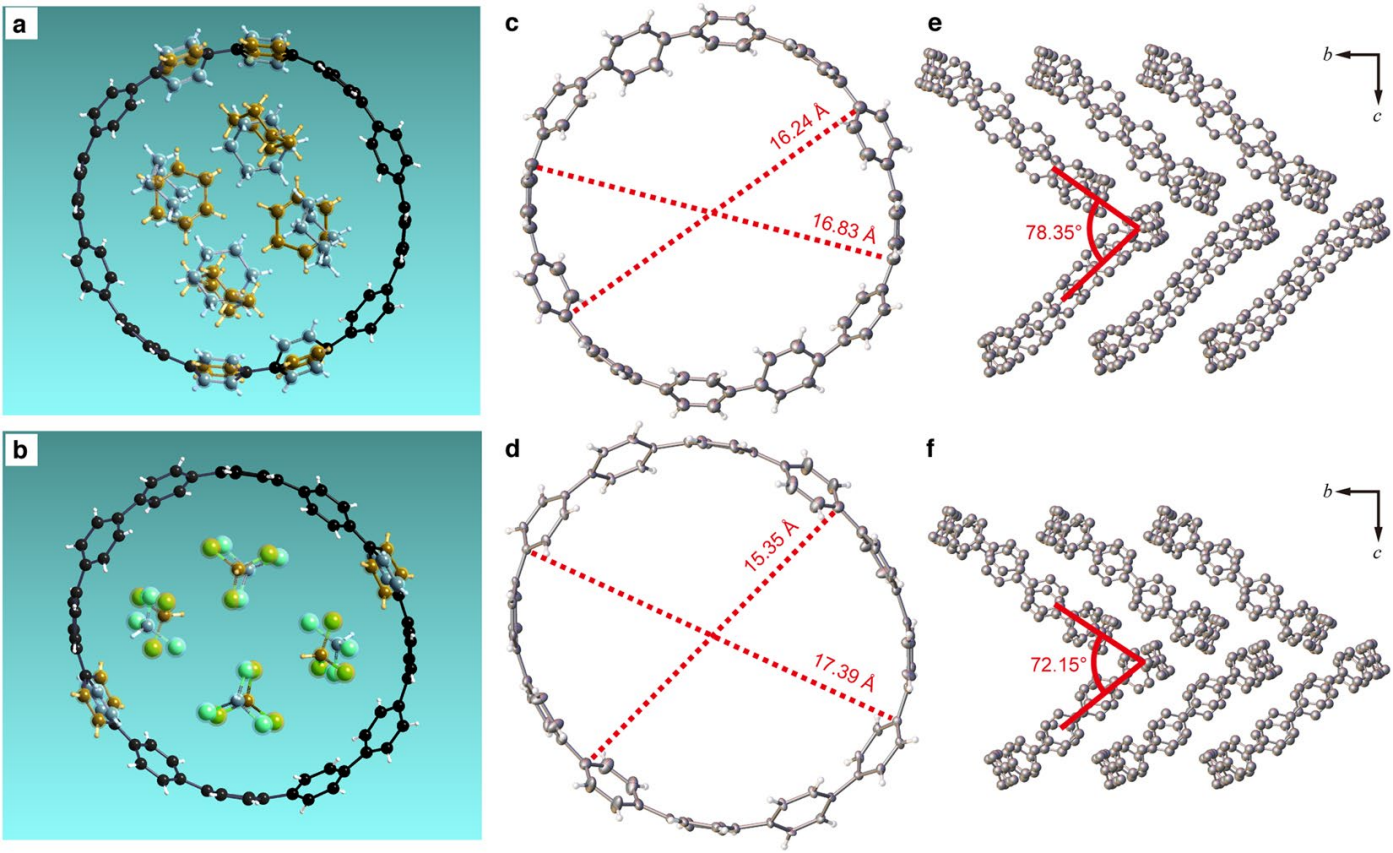
Molecular and crystal structures of 2A and 2B. Molecular structures of [12]CPP containing cyclohexane (**2A**) or chloroform (**2B**) are shown in (**a**,**b**); color code: black (carbon), white (hydrogen), and green (chlorine). In **2A**, two benzene rings were disordered with occupancy ratios of 59:41 and 51:29. The site involving two solvent molecules was also disordered with an occupancy ratio of 60:40. In **2B**, occupancy ratios of 72:28 and 70:30 were observed for the disordered benzene ring and the solvent site, respectively. Disordered sites are depicted by covering atoms with semi-transparent orange and cyan ellipsoids in (**a**,**b**), respectively. ORTEP diagrams of [12]CPP in **2A** (**a**) and **2B** (**b**) are drawn at 50% probability. The longest and shortest distances between symmetrically-related *ipso* carbon atoms in [12]CPP are indicated by red dotted lines. The herringbone packing structure in crystals of **2A** (**c**) and **2B** (**d**) is viewed from the direction perpendicular to the *bc* plane. Dihedral angles between the mean planes of all *ipso* carbon atoms in [12]CPP are shown in red. The solvent molecules contained in the crystal structures have been omitted in (**c**–**f**) for clarity.

As an example for the prior evaluation of a to-be-observed guest molecule based on estimated 〈*B*〉 values, we selected a crystalline host-guest system, specifically, a single crystal of [12]cycloparaphenylene ([12]CPP; **2** in Fig. 1). Molecules of **2** exhibit a macrocyclic structure and crystalize in a porous scaffold structure that hosts solvent molecules as guests^28^. A characterization of the crystal lattice of **2** by powder X-ray diffraction analysis indicated that, although guest exchanges are accompanied by a small rearrangement of **2**, its crystal structure remains essentially isomorphic^29^. In the present study, we grew two single crystals of **2** from a solution of 1 mg of **2** dissolved in chloroform (200 μL) that was treated with a few drops of either cyclohexane (**2A**; reported procedure^28^) or water (**2B**) in order to demonstrate that possibly different guest molecules can be distinguished prior to a full crystal structure analysis. Almost the same number of diffraction data points [**2A**: 391 data points (Supplementary Data 2); **2B**: 360 data points from a resolution up to sin*θ*/*λ* = 0.65 Å^−1^ (Supplementary Data 3) were collected in preliminary measurements (the calculated *S*s of **2A** and **2B** are summarized in Supplementary Table 2). The isomorphism of **2A** and **2B** was confirmed from their lattice parameters, which were determined using the preliminary data sets. A selected *S* in the shell of 0.090 < sin*θ*/*λ* ≤ 0.100 Å^−1^, where *S* was unavailable in the preliminary data of **2A**, was eliminated in the preliminary data of **2B** in order to unify the contribution from core and valence electrons to the estimated 〈*B*〉 values of the two data sets. Bayesian inference using the preliminary data sets for **2A** and **2B** provided 〈*B*〉 values of 3.50 Å^2^ and 3.13 Å^2^, respectively. This difference of 〈*B*〉 between the isomorphic crystals of the same host molecule suggests that distinguishable guest structures in the pores of **2A** and **2B** may potentially be observed and thus encourages the collection of a full data set and carrying out a crystal structure analysis to characterize the unknown host-guest system in **2B**. The crystal structure analysis confirmed our expectation regarding distinguishable host-guest structures, i.e., we observed the enclosure of four molecules of cyclohexane (**2A**) or four molecules of chloroform (**2B**) per molecule of **2** (Fig. 2a,b). Additionally, an elliptic structural distortion of **2** was found for **2B** (Fig. 2c,d), which is clearly reflected in the range of distances between two diametrally opposed *ipso* carbon atoms, whereby the second one is symmetry-generated due to the presence of an inversion center in **2A** (16.25–16.83 Å) and **2B** (15.35–17.39 Å). These distances are summarized in Supplementary Table 3. The calculated volume of cyclohexane (120.04 Å^3^) and chloroform (58.88 Å^3^) indicates that the distortion of **2** in **2B** originates from packing forces and reduces the solvent-accessible space in the ring. Together with the ring deformation, the herringbone packing of **2** is compressed along the *c* axis by enclosure of chloroform molecules [dihedral angles: 78.35° (**2A**) and 72.15° (**2B**); Fig. 2e,f]. In earlier studies^28,29^, **2** was proposed as a shape-persistent molecule owing to the rigid sp^2^-hybridized C–C bonds^30,31^. In the present study, we initially verified the structural softness of **2**, which arises from the co-existence of solvent molecules in the crystal, and that is common in metal-organic frameworks^32^. The prior evaluation of the to-be-observed structure by Bayesian inference should help characterizing and rationalizing functionality in a host-guest system by prior examination of the enclosure space and the exchange with guest molecules.

### Choosing the best exposure time for precise observations

In diffraction measurements for crystal structure analysis, the exposure time for the collection of diffraction data sets is usually chosen based on *I* and its uncertainty (*σ*), which originates from measurement errors that involve scattered and fluorescence X-rays as well as background noise in the detector. All of these errors are usually recorded within a preliminary data set. The expected *I*/*σ* ratio for prolonged or shortened exposure time is often calculated in order to consider the potential magnitude of the errors associated with the to-be-collected diffraction intensities. We also applied the method presented herein to find a *I*/*σ* ratio that promises a suitable precision of the to-be-collected data for the crystal structure analysis under given measurement conditions.

**Figure 3:**
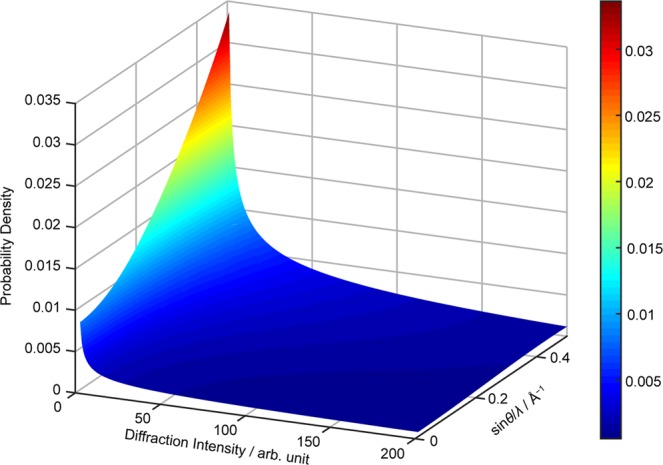
Probability distribution of the diffraction intensities of 3 in the resolution range of the preliminary measurement. The three-dimensional distribution was generated using Eq. (2), in which *S* at a given resolution shell was calculated using Eq. (3), and the estimated value of 〈*B*〉. The surface was drawn by interpolation between the two-dimensional distribution at each resolution. A distribution involving a larger diffraction intensity region is shown in Supplementary Fig. 2.

As the subject for this demonstration, we selected a typical molecular crystal taurine (**3** in Fig. 1). We estimated a 〈*B*〉 value of 1.53 Å^2^ for **3** using the method presented herein based on a preliminary data set up to sin*θ*/*λ* = 0.500 Å^−1^ (the calculated *S*s are summarized in Supplementary Table 4; the corresponding exclusion of *S*s based on the extinction effect is described in the Supplementary Note). The probability distribution of to-be-collected diffraction intensities was derived from Eqs (2) and (3) using the estimated 〈*B*〉 value under consideration of the centrosymmetric nature of **3** (space group assigned based on the preliminary data: *P*2_1_/*c*). The three-dimensional probability distribution for to-be-collected diffraction intensities is shown in Fig. 3 and Supplementary Fig. 2. Here, the probability for intensity overlaps (two or more intensities may have the same amplitude within a range of uncertainty, see also the *Methods* section) was considered using the derived distribution. The highest probability for intensity overlaps was 4.62 × 10^−5^, which was obtained from squaring the probability of the weakest intensity (0 ≤ *I* ≤ 0.01 on a relative scale) at the highest resolution shell (0.490 < sin*θ*/*λ* ≤ 0.500 Å^−1^), where the probability is maximal in the obtained probability distribution. This result suggests that intensity overlaps are scarce in nature and that *I*/*σ* ratios that favor the absence of intensity overlaps are preferable.

**Figure 4:**
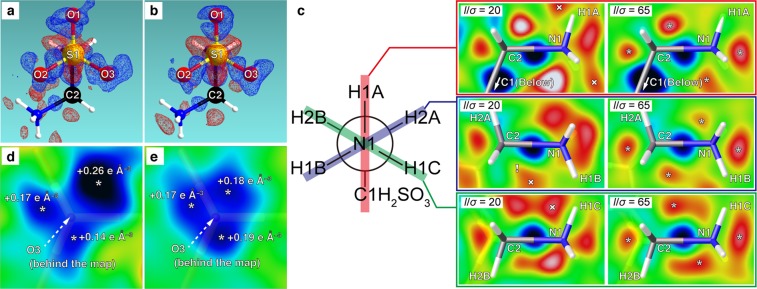
Deformation of the electron density from a spherical distribution in each atom. Positive (blue) and negative (red) density mapped onto 2*σ* isodensity surfaces in (**a**) (*I*/*σ* = 20) become sharply defined in (**b**) (*I*/*σ* = 65). These represent deformation of electron densities around the sulfur atom (S1) from the non-bonding to the bonding positions by sp^3^ hybridization. Electron density decreasing from the non-bonding positions by sp^3^ hybridization (marked by ‘*’) are also clearly observed around the nitrogen (N1) and carbon (C2) atoms in the H–N–C–C and H–N–C–H mean planes (shown in a Newman projection) in (**c**). Inadequate (×) and disappeared (!) density peaks are found in the maps of *I*/*σ* = 20 due to the increased level of noise density. Positive densities from the three lone pairs on the oxygen atom O3 also become clearly recognizable in the plane perpendicular to the S1 = O3 bond and 0.15 Å above O3 by going from *I*/*σ* = 20 (**d**) to *I*/*σ* = 65 (**e**). Positive peak positions are marked by ‘*’ with the corresponding amplitude. The color scheme and the density limit (blue: positive; red: negative; green: zero; ±0.18 e Å^−3^) are used in (**c**–**e**).

The intensity overlaps for a given *I*/*σ* ratio were preliminary evaluated using to-be-collected intensities generated from the derived probability density distribution by Markov chain Monte Carlo methods, which is an algorithm for random sampling according to a given probability distribution^33^. The probability density distribution for the highest resolution shell was used for the sampling, as the *I*/*σ* ratio for this shell is the minimum threshold for the expected data due to the decreasing nature of diffraction intensity with increasing resolution^22^. The sampled intensities and *σ* values calculated for satisfying *I*/*σ* = 20 or 65 are summarized in Supplementary Table 5. The ratios between observed and expected overlapping intensities were 10/28 (*I*/*σ* = 20) and 1/28 (*I*/*σ* = 65). The results of the crystal structure analysis using diffraction data sets collected for *I*/*σ* = 20 or 65 (average) at the evaluated resolution shell are shown in Fig. 4 (electron density) and Supplementary Fig. 3 (molecular structures). By incrementing the X-ray exposure time to satisfy *I*/*σ* = 65, vastly adequate symmetrical deformation of electron density representing sp^3^ hybridization was successfully observed around the sulfur atom (S1 in Fig. 4a,b). Even for the light atoms, such as carbon (C2) and nitrogen (N1) in Fig. 4c, this precision improvement in differential electron density contributed to the identification of sp^3^ hybridization from the observation of negative electron density peaks, some of which were indistinguishable from noise peaks or unobservable for *I*/*σ* = 20. The representation of the lone pairs on the oxygen atoms O1, O2, and O3 was also improved by this increase of exposure time and, especially around O3, a highly precise distribution of three lone pairs around the ideal positions was observed (O3: Fig. 4d,e; others: Supplementary Fig. 4). Choosing an X-ray exposure time that satisfies *I*/*σ* = 20 at a high-resolution shell may be seen empirically as sufficiently high for a precise crystal structure analysis. However, the present method demonstrates the benefits of increasing the exposure time in order to reduce the number of overlapping intensities. A choice of exposure time according to these guidelines will promote the efficiency for the precise observation of electron density distribution of **3** and thus avoid the time-consuming and laborious repetition of trial-and-error measurements.

## Conclusions

We have proposed a method for the evaluation of to-be-collected diffraction data prior to collecting full diffraction data sets and carrying out laborious crystal structure analyses. This method is based on the use of Bayesian inference to estimate 〈*B*〉 values using only a small diffraction data set that can be collected within a short time. The accuracy of the estimated 〈*B*〉 values was confirmed to be comparable to those determined by a conventional crystal structure analysis. Potential applications of this method were demonstrated in the context of the prior structural evaluation of a host-guest system, and considerations regarding the choice of exposure times that promise sufficiently high precision of the resolved structure. Despite the fact that crystal structure analysis is routinely used in many scientific areas, the structural characterization of host-guest systems remains problematic due to e.g. difficulties associated with the growth of suitable crystals and the analysis of the disordered structures of guest molecules. Even though a recent state-of-the-art X-ray diffractometer is used, a day or a longer time is sometimes required to complete data collection due to a weakly-diffracting nature of crystal containing severely-disordered guest molecules. Evaluation of guest exchange based on crystal structures may become laborious due to arduous crystal structure analysis of disordered model. Accompanying recent improvements for the generation of suitable single crystals of porous covalent organic materials^34^, the method presented herein is expected to help improving the throughput of solid-state structural studies of host-guest systems by means of identifying properly guest-exchanged single crystals. The precise determination of the distribution of electron density is one of the most fundamental applications of crystal structure analysis. Quantitative diffraction intensities are indispensable for an in-depth evaluation of electron density distribution of a target molecule by crystal structure analysis^35^. However, data collection for such precise crystallographic observations is not trivial and requires a high level of expertise^36^. While recent programs for the collection of diffraction data can help calculating exposure times to satisfy a given *I*/*σ* ratio, researchers still empirically decide on a target value for the *I*/*σ* ratio for their measurement. Owing to calculation of the exposure time to satisfy the *I*/*σ* ratio provided for collecting a less ‘intensity-overlapping’ data set by the present method, crystallographic observation of orbital hybridization and valence electrons in the differential electron density map will become viable for non-specialists without having to resort to time-consuming and expensive series of diffraction experiments in order to reach results of sufficient precision.

The style of X-ray diffraction data collection is rapidly changing nowadays by development of diffractometer equipped with high-intensity X-ray sources and packaged software which automatically optimizes measurement condition. However, selection of a suitable crystal for measurement and decision of X-ray exposure time for precise observation still rely on researchers own experiences. The present method is introduced and demonstrated as an idea for addressing the above two experience-depended processes. Further sophistication of measurement for crystal structure analysis is expected by combining this method with present packaged software. Especially, the present method will make a large contribution to improve the measurement efficiency in synchrotrons or XFELs. Quick decision of a subject crystal and data accumulation time by the present method will be useful for minimizing sample deterioration due to radiation damage on further collection of a full data set. Because the present method is a fundamental technology, wide range of application of it for helping crystal structure analysis is expected.

## Methods

### X-ray crystal structure analysis

All crystal structures were solved by dual-space methods (*SHELXT*^10^) and refined using the full-matrix least-squares method (*SHELXL*^9^). Detail of data collections and analyses are described in the Supplementary Materials and Methods.

### Density functional theory (DFT) calculations to obtain volumes of solvent molecules

The geometries of cyclohexane and chloroform were optimized by DFT calculations at the B3LYP/cc-pVDZ level of theory. The program Gaussian 09^37^ was used for the geometrical optimizations. Input geometries were built using the program GaussView^38^. Optimized geometries were confirmed as energetic minima by vibrational analysis at the same level of theory. The volume of 1 mol of the molecules was obtained as the output of the calculations and divided it by the Avogadro number to obtain the volume for one molecule.

### Evaluation of to-be-collected diffraction intensities by considering intensity overlapping

The number of the to-be-collected intensities at a selected resolution shell was counted in the output of the generated diffraction data based on defined lattice parameters and the space group of **3** (HKLF4 GENERATE function in PLATON^14^). At the shell 0.490 < sin*θ*/*λ* ≤ 0.500 Å^−1^, 28 of the expected diffraction data points were found for collection. A distribution function of diffraction intensities at this resolution shell was obtained from Eq. (2), in which the parameter *Σ* was calculated from Eq. (3) using the estimated 〈*B*〉 and *f*_*j*_s calculated from the following equation:

7 $$\begin{array}{c}f=\mathop{\sum }\limits_{i=1}^{4}{a}_{i}\,\exp \{\,-\,{b}_{i}{(\sin \theta /\lambda )}^{2}\}+c.\end{array}$$

The coefficients *a*_*i*_ and *b*_*i*_ (*i* = 1–4) as well as *c* for each atom are tabulated in the *International Tables for Crystallography*^39^. Sampling of 28 intensities from the derived distribution function using Markov chain Monte Carlo methods was performed by the slice sampler^40^ implemented in the program MATLAB (The MathWorks, Inc., USA). Uncertainties of all sampled intensities were calculated to satisfy *I*/*σ* = 20 or 65. The sampled intensities were arranged in decreasing order to evaluate any potential intensity overlapping. When the difference between two consecutive intensities was less than the sum of their uncertainties, overlap occurred.

## Supplementary information

Supplementary Information

Supplementary Data 1

Supplementary Data 2

Supplementary Data 3

Supplementary Data 4

Supplementary Data 5

Supplementary Data 6

Supplementary Data 7

Supplementary Data 8

Supplementary Data 9

Supplementary Data 10

Supplementary Table 1

Supplementary Table 2

Supplementary Table 3

Supplementary Table 4

Supplementary Table 5

## Data Availability

Crystallographic data together with diffraction intensities (also used for the estimation by Bayesian inference) reported in this paper have been deposited at the Cambridge Crystallographic Data Centre (CCDC) under reference numbers (1878473–1878479). All data are available in in the main text or the Supplementary data 1 to 10, and/or from the corresponding author on request.
