# Supplementary material for: Inference-assisted intelligent crystallography based on preliminary data
Source: Sci Rep. 2019 Aug 22;9:11886. doi: 10.1038/s41598-019-48362-3 (PMC6706436; doi:10.1038/s41598-019-48362-3)
Supplement: Supplementary file 1 — Supplementary Information [file 41598_2019_48362_MOESM1_ESM.pdf]

[Supplementary Information]

Inference-assisted intelligent crystallography based on preliminary data

Manabu Hoshino[1,2]*, Yoshinori Nakanishi-Ohno[1,3,4], Daisuke Hashizume[2]

[1]PRESTO, Japan Science and Technology Agency (JST), 4-1-8 Honcho, Kawaguchi, Saitama, 332-0012, Japan.

[2]RIKEN Center for Emergent Matter Science (CEMS), 2-1 Hirosawa, Wako, Saitama, 351-0198, Japan.

[3]Graduate School of Arts and Sciences, The University of Tokyo, 3-8-1 Komaba, Meguro, Tokyo, 153-8902, Japan.

[4]Komaba Institute for Science, The University of Tokyo, 3-8-1 Komaba, Meguro, Tokyo, 153-8902, Japan.

*email: manabu.hoshino@riken.jp

This file includes:

Supplementary Materials and Methods
Supplementary Notes
References 41 to 44
Supplementary Figs. 1 to 6

Supplementary Materials and Methods

X-ray diffraction measurement and crystal structure analysis of 2-dimethylsufuranylidene-1,3-indanedione (1). The crystal structure of **1** has been reported as non-centrosymmetric[41]. The retrieval of the phase for diffraction intensities of non-centrosymmetric crystals is usually difficult, as any phase angle may be assigned arbitrarily to each intensity[22]. Therefore, **1** was selected as the first example to demonstrate the efficiency of the method presented herein by circumventing the phase problem. For that purpose, a single crystal of **1** [0.49 × 0.40 × 0.34 mm^3, provided by Rigaku (Japan) in order to check the diffractometer] was used for the measurement. The crystal was mounted on a diffractometer (SuperNova with EOS S2 CCD detector; Rigaku, Japan) and a preliminary diffraction data set (used as the subject data set) was collected. Subsequently, the diffraction data set for the crystal structure analysis (reference data set) was collected. Both data collections were performed using X-rays of Mo$K\alpha$ radiation. The subject crystal was cooled to 100 K on a diffractometer using a cold nitrogen stream. The intensity integration, numerical and empirical absorption correction, and scaling for the collected data sets were processed using the program CrysAlisPro (version 1.171.39.46; Rigaku, Japan). In crystal structure analyses, all non-hydrogen atoms were refined anisotropically. All hydrogen atoms were refined isotropically without any restraint and constraint according to their coordinates and an isotropic thermal factor (U_{iso}). Extinction effects were evaluated by applying the EXTI command in *SHELXL*, and the program suggested a significant correction coefficient [0.014(3)]. The averaged isotropic thermal parameter ($\langle B\rangle$) was calculated as $\langle B\rangle$ = 1.82(46) Å^2 from U_{iso}s for all atoms listed in the crystallographic information file (CIF) using the following equation:

$$\langle B\rangle = 8\pi^2\langle U_{iso}\rangle. \qquad (8)$$

A refined formula and the weight (M_r) of $C_{11}H_{10}O_2S$ and 206.25, respectively were obtained. The space group was assigned as $P2_12_12_1$. For the structural analysis, 2178 unique out of 4015 recorded reflections (3.193 < θ < 27.479°) were used (R_{int} = 0.0257). Cell constants, an R-factor on $F^2 > 2\sigma(F^2)$, and a weighted R-factor of a = 5.8358(2) Å, b = 8.9119(3) Å, c = 18.2739(6) Å, V = 950.39(6) Å^3, R-factor on $F^2 > 2\sigma(F^2)$: = 0.0336, weighted R-factor: wR = 0.0766, and S = 1.054 were obtained. A density of 1.441 g cm^{-3} was calculated. A linear absorption coefficient (μ) was given as 0.305 mm^{-1}. A residual electron density (max/min) of 0.256/–0.315 e Å^{-3} was calculated, while the Flack parameter [0.07(6)] was refined according to the Parsons method[42].

The crystal structure analysis using the excluded reference data set (described in the main manuscript and Supplementary Materials) was performed as described above: $\langle B\rangle$ = 1.82(50) Å^2; 1992 unique out of 3560 recorded reflections (4.144 < θ < 27.479°) were used (R_{int} = 0.0272); cell constants: a = 5.8358(2) Å, b = 8.9119(3) Å, c = 18.2739(6) Å, and V = 950.39(6) Å^3; R-factor on $F^2 > 2\sigma(F^2)$: R =

0.0346; weighted R-factor: $wR = 0.0771$; $S = 1.068$; calculated density: 1.441 g cm^{-3}; linear absorption coefficient (μ): 0.305 mm^{-1}; residual electron density (max/min): 0.233/–0.297 e Å^{-3}; Flack parameter refined by the Parsons method: 0.09(6); coefficient suggested by the EXTI command: 0.012(4).

X-ray diffraction measurement and crystal structure analysis of [12]CPP·4(cyclohexane) (2A) and [12]CPP·4($CHCl_3$) (2B) ([12]CPP: [12]cycloparaphenylene, 2). A powder of **2** was purchased from TCI (Japan) for recrystallization. Single crystals of **2A** (0.15 × 0.14 × 0.06 mm^3) and **2B** (0.18 × 0.15 × 0.05 mm^3) were used as subjects. The crystals were cooled to 100 K using a cold nitrogen stream. Preliminary and full data sets of **2A** and **2B** were collected on a diffractometer (SuperNova with EOS S2 CCD detector; Rigaku, Japan) using X-rays of Cu$K\alpha$ radiation. In order to obtain significant intensities from solvated crystals at high angles, which generally diffract weakly, the collection time of the preliminary data of **2A** (5.625 min in total) and **2B** (11.25 min) was prolonged compared to that of **1**. During a check of the preliminary data of **2B**, twinning of the crystal was implied by the positions of diffraction spots in reciprocal space. The twinning of **2B** was confirmed by careful examination of a full data set in reciprocal space and the diffraction data sets of each twinned phase were separately processed in a data integration process. Data processing (integration, correction, and scaling) was conducted as described for **1**. For **2B**, the data set of a phase that provided a better R_{int} value (R_{int} = 0.0663; for the other phase: R_{int} = 0.1708) was selected for a further crystal structure analysis. In crystal structure analyses, all non-hydrogen atoms were refined anisotropically. All hydrogen atoms were refined isotropically using the riding model and proper AFIX commands. The geometry of the cyclohexane molecules in **2A** was refined by applying restrictions to the ideal bond lengths and angles via DFIX and DANG commands. Chloroform molecules in **2B** were restricted to a similar geometry in the refinement (SAME command). Anisotropic thermal parameters of all solvent molecules in both crystals were refined as approximately isotropic (ISOR command) and to have the same parameters as the connected atoms (the SIMU command). Due to the restrictions toward U_{iso} of the hydrogen atoms in **2A** and **2B**, a proper calculation of $\langle B \rangle$ from the result of crystal structure analysis was impossible.

2A: $C_{72}H_{48}$·4($C_{24}H_{48}$); M_r = 1249.72; space group: $P2_1/c$; 5559 unique out of 23028 recorded reflections ($3.935 < \theta < 73.428°$) were used (R_{int} = 0.0406); cell constants: a = 18.5711(7) Å, b = 8.2168(3) Å, c = 23.3721(10) Å, β = 105.990(4)°, V = 3428.5(2) Å^3; R-factor on $F^2 > 2\sigma(F^2)$: R = 0.1134; weighted R-factor: wR = 0.3447; S = 1.470. Calculated density: 1.211 g cm^{-3}; linear absorption coefficient (μ): 0.507 mm^{-1}; residual electron density (max/min): 1.048/–0.642 e Å^{-3}.

2B: $C_{72}H_{48}$·4($CHCl_3$); M_r = 1390.57; space group: $P2_1/c$; 3031 unique out of 10299 recorded reflections ($4.316 < \theta < 67.078°$) were used (R_{int} = 0.0663); cell constants: a = 19.1151(12) Å, b = 8.3459(5) Å, c = 20.9986(10) Å, β = 102.637(6)°, V = 3268.8(3) Å^3; R-factor on $F^2 > 2\sigma(F^2)$: R =

0.1494; weighted R-factor: $wR = 0.4020$; $S = 1.515$; calculated density: 1.413 g cm^{-3}; linear absorption coefficient (μ): 5.003 mm^{-1}; residual electron density (max/min): 1.976/–1.010 e Å^{-3}.

X-ray diffraction measurement and crystal structure analysis of 2-aminoethanesulfonic acid (taurine, 3). A powder of **3** was purchased from Nakarai (Japan) and crystals were obtained from the slow evaporation of a saturated aqueous solution of **3**. A single crystal of **3** (0.131 × 0.063 × 0.046 mm^3) was cooled to 100 K using a cold nitrogen stream. A single-crystal X-ray diffractometer equipped with a two-dimensional photon counting detector (Synergy-i with HyPix Bantum detector; Rigaku, Japan) was used for the collection of diffraction data sets.

After collection of the preliminary data of **3** ($\sin\theta/\lambda < 0.50$ Å^{-1}; data accumulation time: 0.625 min), diffraction data sets satisfying $I/\sigma = 20$ and 65 (average) at the shell of 0.490 Å^{-1} < $\sin\theta/\lambda \leq 0.500$ Å^{-1} were collected consecutively. Data processing (integration, correction, and scaling) was conducted as for the samples above. In crystal structure analyses, all non-hydrogen atoms were refined anisotropically. All hydrogen atoms were refined isotropically without any restraint and constraint for their coordinates and U_{iso}. Coefficients for extinction effects were considered by applying the EXTI command in the refinement [$I/\sigma = 20$: 0.0069(10); $I/\sigma = 65$: 0.0070(8)]. Calculated $\langle B \rangle$s values of 1.19(4) Å^2 and 1.21(3) Å^2 were calculated for $I/\sigma = 20$ and $I/\sigma = 65$, respectively; $C_2H_7NO_3S$; M_r = 125.15; space group: $P2_1/c$.

Crystal structure analysis of **3** ($I/\sigma = 20$): 834 unique out of 1972 recorded reflections ($6.857 < \theta < 69.714°$) were used ($R_{int} = 0.0188$); cell constants: a = 5.2633(1) Å, b = 11.6313(2) Å, c = 7.7812(1) Å, β = 93.982(2)°, V = 475.207(14) Å^3; R-factor on $F^2 > 2\sigma(F^2)$: $R = 0.0282$; weighted R-factor: wR = 0.0717; S = 1.071; calculated density: 1.749 g cm^{-3}; linear absorption coefficient (μ): 5.242 mm^{-1}; residual electron density (max/min): 0.458/–0.370 e Å^{-3}.

Crystal structure analysis of **3** ($I/\sigma = 65$): 895 unique out of 4862 recorded reflections ($6.853 < \theta < 69.969°$) were used ($R_{int} = 0.0139$); By improvement of I/σ, some weak intensities, which were below the level of the measurement error and eliminated in the refinement for $I/\sigma = 20$, became significant and were involved in the unique reflections for refinement. Cell constants: a = 5.2632(1) Å, b = 11.6307(1) Å, c = 7.7882(1) Å, β = 94.004(1)°, V = 475.589(12) Å^3; R-factor on $F^2 > 2\sigma(F^2)$: R = 0.0209; weighted R-factor: $wR = 0.0549$; $S = 1.126$; calculated density: 1.748 g cm^{-3}; linear absorption coefficient (μ): 5.239 mm^{-1}; residual electron density (max/min): 0.381/–0.331 e Å^{-3}.

Supplementary Note

Implementation of Bayesian inference for estimating $\langle B \rangle$. The preliminary collected diffraction data sets after intensity integration, correction, and scaling as described in the *Supplementary Materials and Methods* section were used for the estimation of $\langle B \rangle$ by Bayesian inference. The probability

distribution of $\langle B\rangle$ of a subject crystal is given by the posterior distribution calculated using equation (5). The calculated $\langle I\rangle$s from the collected diffraction intensities in each resolution shell is substituted for Σ in equation (4) while approximating $\langle I\rangle$ to Σ. For the data collected using Cu$K\alpha$ radiation (λ = 1.54148 Å), the shell width was set to 0.01 Å^{-1}, which is commonly used for tabulating atomic scattering factors in the *International Tables for Crystallography*[39]. Half the width was applied for the data collected using Mo$K\alpha$ radiation (λ = 0.71073 Å) in order to average intensities lying on a comparable θ range for both X-ray sources. The center value of a resolution shell was substituted for $\sin\theta/\lambda$ in the following calculations. The thus obtained Σ was divided by the sum of f_j^2 to calculate S. In this calculation, f_js are obtained from equation (7).

The right side of equation (4) is normalized to describe $P(\boldsymbol{S}|\langle B\rangle)$ in equation (5) using the result of the following calculation:

$$\int_{-\infty}^{\infty} \exp\{-2\langle B\rangle(\sin\theta/\lambda)^2\}\, d(\sin\theta/\lambda) = \left(\frac{\pi}{2\langle B\rangle}\right)^{\frac{1}{2}}. \quad (9)$$

As the integration of a probability distribution function over the entire space should be equal to one, i.e., the sum of the probability for all events should be 100%, the right side of equation (4) was divided by $(\pi/2\langle B\rangle)^{1/2}$ in order to normalize it as a probability density distribution function. The likelihood for S at the mth shell $[P(S_m|\langle B\rangle)]$ is described by multiplying the above probability distribution function S_m times. Here, we omitted the normalization of $P(S_m|\langle B\rangle)$ and their product $[P(\boldsymbol{S}|\langle B\rangle)]$, as $P(\langle B\rangle|\boldsymbol{S})$ is given by the proportional expression of equation (5). The proportionality symbol ($\propto$) in equation (6) is used to express this omission. All distributions of the calculated $P(\langle B\rangle|\boldsymbol{S})$ are shown in Supplementary Fig. 5.

In the present method, the value of $\langle B\rangle$ that provided the maximum probability density in $P(\langle B\rangle|\boldsymbol{S})$ was chosen as the estimated value of $\langle B\rangle$ for the subject crystal and obtained by the maximum likelihood method[43]:

$$L(\langle B\rangle) \equiv \left(\frac{2\langle B\rangle}{\pi}\right)^{\sum_{m=1}^{n}\frac{S_m}{2}} \exp\left\{-2\langle B\rangle \sum_{m=1}^{n} S_m(\sin\theta_m/\lambda)^2\right\}$$

$$\log L(\langle B\rangle) = \frac{1}{2}\sum_{m=1}^{n} S_m\{\log 2\langle B\rangle + \log(2/\pi)\} - 2\langle B\rangle \sum_{m=1}^{n} S_m(\sin\theta_m/\lambda)^2$$

$$\frac{\partial \log L(\langle B\rangle)}{\partial\langle B\rangle} = \frac{1}{2\langle B\rangle}\sum_{m=1}^{n} S_m - 2\sum_{m=1}^{n} S_m(\sin\theta_m/\lambda)^2. \quad (10)$$

Here, $L(\langle B\rangle)$ is defined as the likelihood [$P(\boldsymbol{S}|\langle B\rangle)$] for a simple description. When

$$\langle B\rangle = \left(\sum_{m=1}^{n} S_m\right) \bigg/ \left(4\sum_{m=1}^{n} S_m(\sin\theta_m/\lambda)^2\right), \tag{11}$$

equation (10) equals zero and equation (11) corresponds to the estimated value of $\langle B\rangle$.

Influence of the selection of a prior distribution on the estimated $\langle B\rangle$. The maximum likelihood estimation is applicable to Bayesian inference when a prior distribution is given as a uniform distribution. Here, the estimation is demonstrated for the case of using another distribution function as a prior distribution in equation (5). Because $\langle B\rangle$ is defined as an averaged parameter representing isotropic thermal motion of an atom, a normal distribution function is appropriate to describe our knowledge about $\langle B\rangle$ as a prior distribution;

$$P(\langle B\rangle) = \frac{1}{\sqrt{2\pi\sigma^2}}\exp\left\{-\frac{(\langle B\rangle-\mu)^2}{2\sigma^2}\right\}. \tag{12}$$

Here, μ and σ^2 are the mean and variance of $\langle B\rangle$ in our prior knowledge. The posterior distribution is calculated by

$$\begin{aligned} P(\langle B\rangle|\boldsymbol{S}) &\propto P(\boldsymbol{S}|\langle B\rangle)P(\langle B\rangle) \\ &= \frac{1}{\sqrt{2\pi\sigma^2}}\left(\frac{2\langle B\rangle}{\pi}\right)^{\sum_{m=1}^{n}\frac{S_m}{2}}\exp\left\{-2\langle B\rangle\sum_{m=1}^{n} S_m(\sin\theta_m/\lambda)^2 - \frac{(\langle B\rangle-\mu)^2}{2\sigma^2}\right\}. \end{aligned} \tag{13}$$

As $P(\langle B\rangle|\boldsymbol{S})$ is a deformed bell-shaped function, $\langle B\rangle$, which is provided as the maximum probability density [obtained from the so-called maximum *a posteriori* (MAP) estimation], was calculated from the first derivative of equation (13), similar to the maximum likelihood method:

$$\log P(\langle B\rangle|\boldsymbol{S}) = \log\left(\frac{1}{\sqrt{2\pi\sigma^2}}\right) + \frac{1}{2}\sum_{m=1}^{n} S_m\left\{\log 2\langle B\rangle + \log\left(\frac{2}{\pi}\right)\right\}$$
$$- 2\langle B\rangle \sum_{m=1}^{n} S_m(\sin\theta_m/\lambda)^2 - \frac{(\langle B\rangle - \mu)^2}{2\sigma^2}$$
$$\frac{\partial \log P(\langle B\rangle|\boldsymbol{S})}{\partial\langle B\rangle} = \frac{1}{2\langle B\rangle}\sum_{m=1}^{n} S_m - 2\sum_{m=1}^{n} S_m(\sin\theta_m/\lambda)^2 - \frac{\langle B\rangle - \mu}{\sigma^2}. \tag{14}$$

When our prior knowledge of $\langle B\rangle$ is obscure, σ^2 in equation (14) is substituted with a large value, which renders the third term on the right side of this equation negligible. Consequently, the estimated $\langle B\rangle$ given from the condition that equation (14) equals zero can be regarded as equivalent to equation (11). In the present method, the maximum likelihood estimation method is used to analytically calculate $\langle B\rangle$ by assuming no prior information is available. When a definite knowledge about $\langle B\rangle$ is applied to equation (14), i.e., σ^2 in equation (14) is substituted with a very small value, the third term of equation (14) becomes dominant and a very close value to μ, given as a prior knowledge, is provided as an estimated value. A uniform distribution was selected as $P(\langle B\rangle)$ in the present study to avoid such an arbitrary estimation.

Effect of data unavailability on the estimation of $\langle B\rangle$ by the present method. The slopes, which represent contributions from the core and valence electrons to an atomic scattering factor as a function of the resolution, are clearly distinguishable[26]: The contribution from valence electrons is negligible and core electrons offer a significant contribution at the high-resolution range. As diffraction is a consequence of interference of scattering waves, the contribution of core and valence electrons to the diffraction intensities shows the same dependence on the resolution. Valence electrons are broadly distributed upon the formation of chemical bonds and intermolecular interactions. On the other hand, core electrons are centered on atomic nuclei. Therefore, crystal structure analyses that use data sets that involve only low-resolution intensities are expected to provide larger $\langle B\rangle$ values on account of the increased contribution from valence electrons compared to crystal structure analyses that use data sets with higher resolution.

Here, the dependence on the resolution of $\langle B\rangle$, obtained from crystal structure analyses that use masked data sets is demonstrated. The two masked data sets were prepared by subjectively eliminating diffraction data lying on a selected resolution range. The reference data set of **1** was used as the original data set for this elimination. The first masked data set contains diffraction data points in the range $0 < \sin\theta/\lambda \leq 0.500$ Å^{-1}, i.e., intensities in the high-resolution region of the original data set are masked. The second contains diffraction data points in the range $0.400 < \sin\theta/\lambda \leq 0.650$ Å^{-1}, i.e., the low-

resolution region was masked. For both masked data sets, an isotropic thermal parameter for all hydrogen atoms could not be freely refined in the crystal structure analysis due to the missing data. Therefore, averaged *B* values for the carbon ($\langle B_C \rangle$), oxygen ($\langle B_O \rangle$), and sulfur (B_S) atoms were compared. The original, the first masked, and the second masked data sets provided the following respective values: $\langle B_C \rangle$: 1.50(6), 1.51(10), and 1.43(7) Å^2; $\langle B_O \rangle$: 1.59(4), 1.66(7), and 1.49(5) Å^2; B_S: 1.26(2), 1.41(2), and 1.12(2) Å^2. Especially in B_S, a significant increase and decrease of *B* were observed upon masking. From this comparison, the above expectation that $\langle B \rangle$ values increase upon masking intensities in the high-resolution range and that $\langle B \rangle$ values decrease upon masking low-resolution intensities, where core electrons dominantly contribute to the data sets, was confirmed.

In analogy, excluding data also changes the estimated value of $\langle B \rangle$ using the present method. Estimated $\langle B \rangle$ values of 2.73 and 0.95 Å^2 were obtained using the first and second masked data sets, respectively. Considering the above discussion, the reason for the difference of $\langle B \rangle$ estimated using the subject and reference data set of **1** (described in the main text) should be the lack of many *S*s at a low-resolution range in the subject data (Supplementary Table 1). It should also be noted that the change of $\langle B \rangle$ estimated by the present method is, due to the missing data, more sensitive than that obtained by the crystal structure analysis. Crystal structure analysis using the excluded reference data set of **1** (described in the *Supplementary Materials and Methods* section) provided $\langle B \rangle$ = 1.82(50) Å^2, which is almost consistent with the value obtained from the (not excluded) reference data set. The ratio of excluded to collected intensities was 436/4015 (~ 11%). On the other hand, the ratio of excluded to calculated *S*s by the collected intensities was 25/110 (~ 23%). These ratios demonstrate the merely moderate influence on $\langle B \rangle$ that originates from the unavailability of data in the crystal structure analysis relative to Bayesian inference.

Limitations of the present method – Exclusion of data biased by extinction effects. The present method is based on Wilson's statistics represented in equation (1). If diffraction intensities do not obey these statistics, an estimation of $\langle B \rangle$ using these is beyond the limits of this method. Taurine (**3**) was selected to demonstrate the limits and a practical strategy for addressing this issue. The previously published precise crystal structure analysis of **3** reports the necessity for a correction of the extinction effects on the diffraction intensities[44]. Extinction effects originate from an attenuation of diffracted X-rays in a crystal, and diffraction data points that suffer from this effect are beyond the scope of Wilson's statistics.

Extinction effects are usually evaluated by structural refinement in crystal structure analysis. In the present study of **3**, extinction effects on the diffraction data were thus evaluated from a brief crystal structure analysis prior to using them for the estimation of $\langle B \rangle$ by Bayesian inference. In the data collection for this brief analysis, the measured resolution range and coverage of intensities compared

to the expected ones in that range was limited to $0.126 < \sin\theta/\lambda \leq 0.500$ Å^{-1} and 91.8%, which is close to the minimum of data points required to execute a crystal structure analysis. Processing of the data was performed in the same way as described in the *Supplementary Materials and Methods* section. In the structural refinement, an isotropic thermal parameter for all hydrogen atoms could not be freely refined due to the restriction of the resolution. Due to the moderate amount of data included, the results of the crystal structure analysis (geometrical and thermal parameters) should only be considered for guidance. The file containing this crystallographic data and diffraction data set was deposited at the CCDC under reference number (1878474).

In *SHELXL* (version 2018/1), the attenuation of the diffraction intensities by extinction effects is corrected using the following equation:

$$I_{c(corr.)} = I_c\left(1 + \frac{0.001 I_c \lambda^3 x}{\sin 2\theta}\right)^{-\frac{1}{2}}. \quad (15)$$

Here, I_c is a calculated diffraction intensity, while x is an extinction parameter refined by crystal structure analysis. The above brief crystal structure analysis provided $x = 0.0109(12)$. In the present study, a threshold of θ for disregarding the extinction effect was set as the angle where the fraction containing a parameter x in equation (15) is less than 0.005%. In the present study, the threshold of θ was set to 27° ($\sin\theta/\lambda = 0.294$ Å^{-1}) and the shells below this threshold were excluded from the data set used for the estimation of $\langle B \rangle$.

Based on the above setting of the threshold, the collected data in the range of $\sin\theta/\lambda < 0.300$ Å^{-1} were excluded from the estimation of $\langle B \rangle$ by Bayesian inference. To practically find a suitable threshold, a Wilson plot was used (Supplementary Fig. 6). This plot shows the logarithm of each S toward $(\sin\theta/\lambda)^2$ in order to visualize the conventional estimation of $\langle B \rangle$ by a fitting to the linear function (described in the main manuscript). In this plot, an apparent decrease of S below the threshold is shown. Practically, this visual assessment using a Wilson plot is proposed to identify a threshold for suppressing the extinction effects in order to properly estimate $\langle B \rangle$ without having to perform a crystal structure analysis.

As described in the *Supplementary Materials and Methods* section, the correction of the extinction effects was also performed in the crystal structure analysis of **1**. The calculated threshold for θ in the reference and the excluded reference data sets was $\theta \sim 3°$. As there is no diffraction data in the resolution range below this threshold, all prepared Ss were used for the estimation.

References

41. Guzei, I. A., Bikzhanova, G. A., Spencer, L. C., Timofeeva, T. V., Kinnibrugh, T. L. & Campana, C. F. Polymorphism and History of 2-Dimethylsufuranylidene-1,3-indanedione (YLID). *Cryst. Growth Des.* **8**, 2411–2418 (2008).

42. Parsons, S., Flack, H. D. & Wagner, T. Use of intensity quotients and differences in absolute structure refinement. *Acta Crystallogr. Sect. B*, 69, 249–259 (2013).

43. Bishop, C. M. *Pattern Recognition and Machine Learning* (Springer, New York, 2006).

44. Hibbs, D. E., Austin-Woods, C. J., Platts, J. A., Overgaard, J. & Turner, P. Experimental and Theoretical Charge Density Study of the Neurotransmitter Taurine. *Chem. Eur. J.* **9**, 1075–1084 (2003).

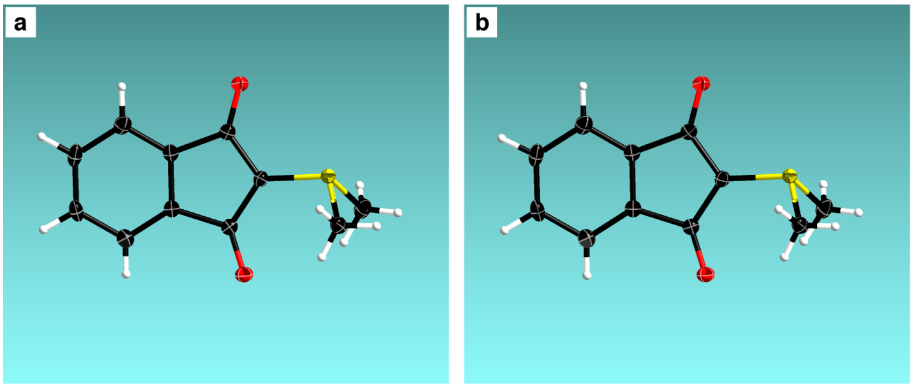

Supplementary Fig. 1| Molecular structure of 1. ORTEP diagrams from the reference (**a**) and the excluded reference (**b**) data sets drawn at 50% probability. Color code: carbon (black), hydrogen (white), oxygen (red), and sulfur (yellow).

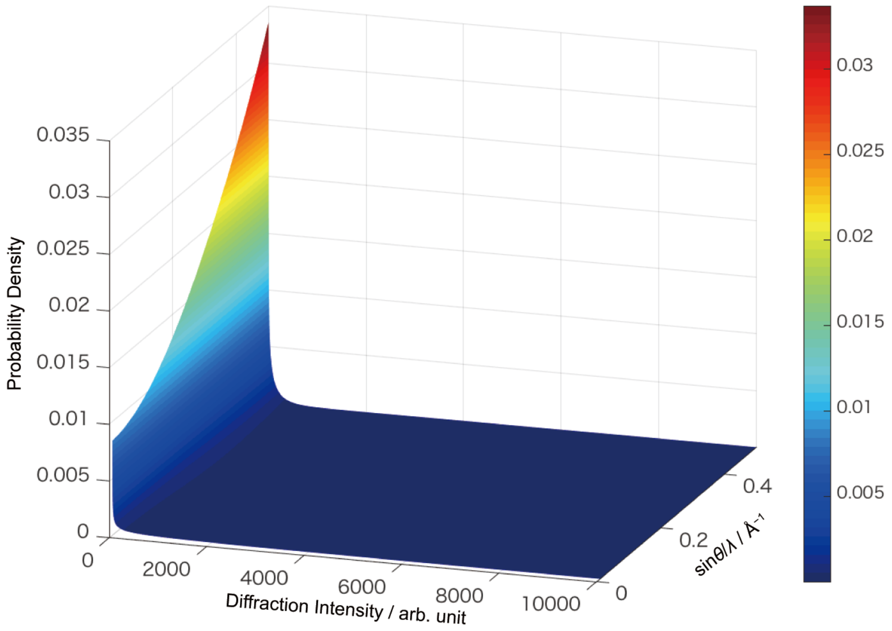

Supplementary Fig. 2| Probability distribution of the diffraction intensities of 3 up to *I* = 10000. The probability density sharply rises approaching I = 0 at all resolution shells. The probability distribution up to I = 200 is shown as Fig. 3 in the main manuscript.

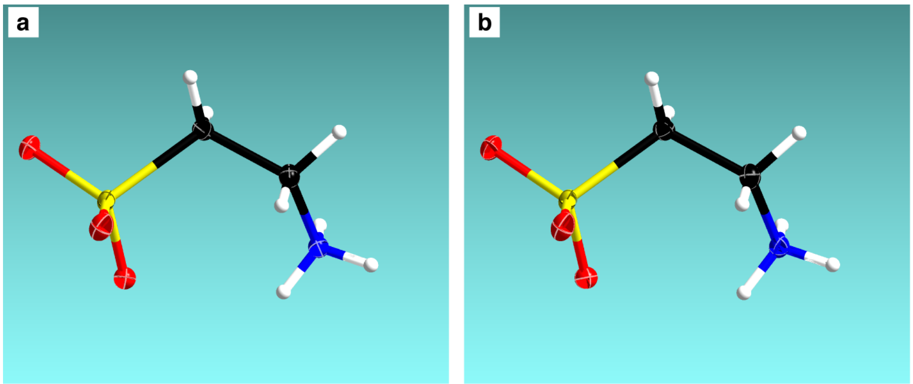

Supplementary Fig. 3| Molecular structure of 3. ORTEP diagrams obtained from the data sets of $I/\sigma = 20$ (**a**) and $I/\sigma = 65$ (**b**) at 50% probability. Color code: carbon (black), hydrogen (white), nitrogen (blue), oxygen (red), and sulfur (yellow).

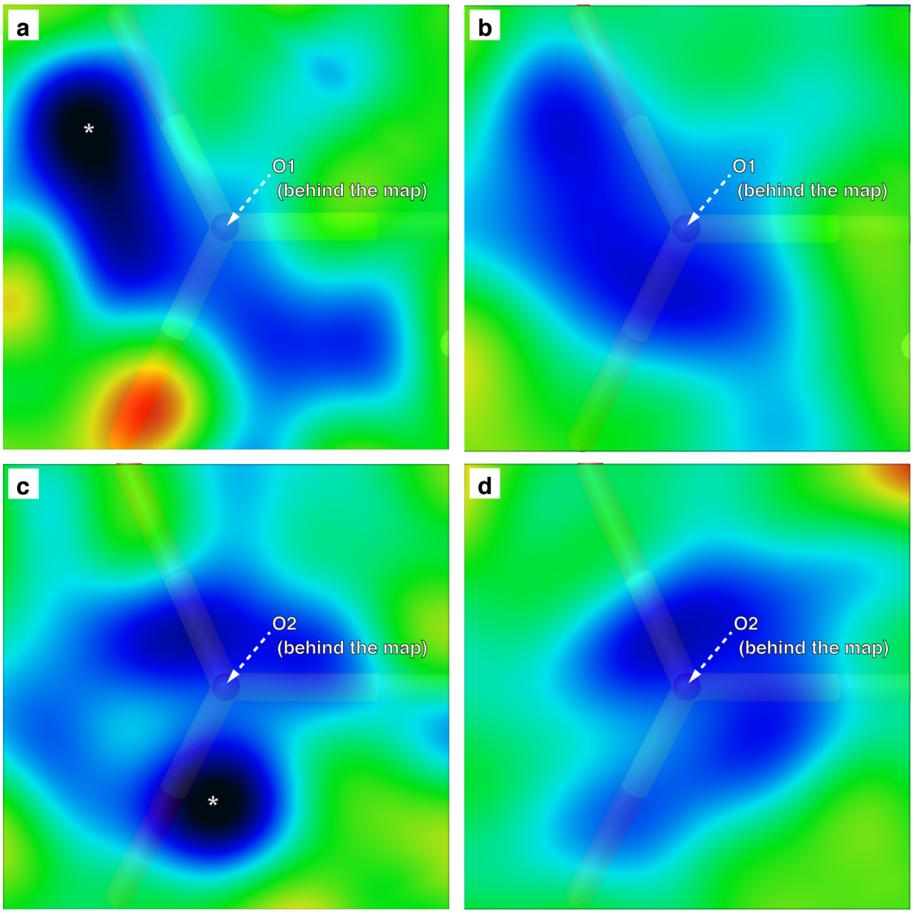

Supplementary Fig. 4| Deformation of electron density from spherical distribution for the selected oxygen atoms. The maps perpendicular to the selected S=O bond and 0.15 Å above the oxygen atom in the bond were drawn. Color code: blue = positive (max. = 0.18 e $Å^{-3}$); red = negative (min. = −0.12 e $Å^{-3}$); green = zero]. Saturated positive density peaks are marked by '*'. Deformation around O1 (**a**: $I/\sigma = 20$; **b**: $I/\sigma = 65$) and O2 (**c**: $I/\sigma = 20$; **d**: $I/\sigma = 65$) is shown. A reduction of noise densities upon increasing I/σ is observed between the corresponding maps.

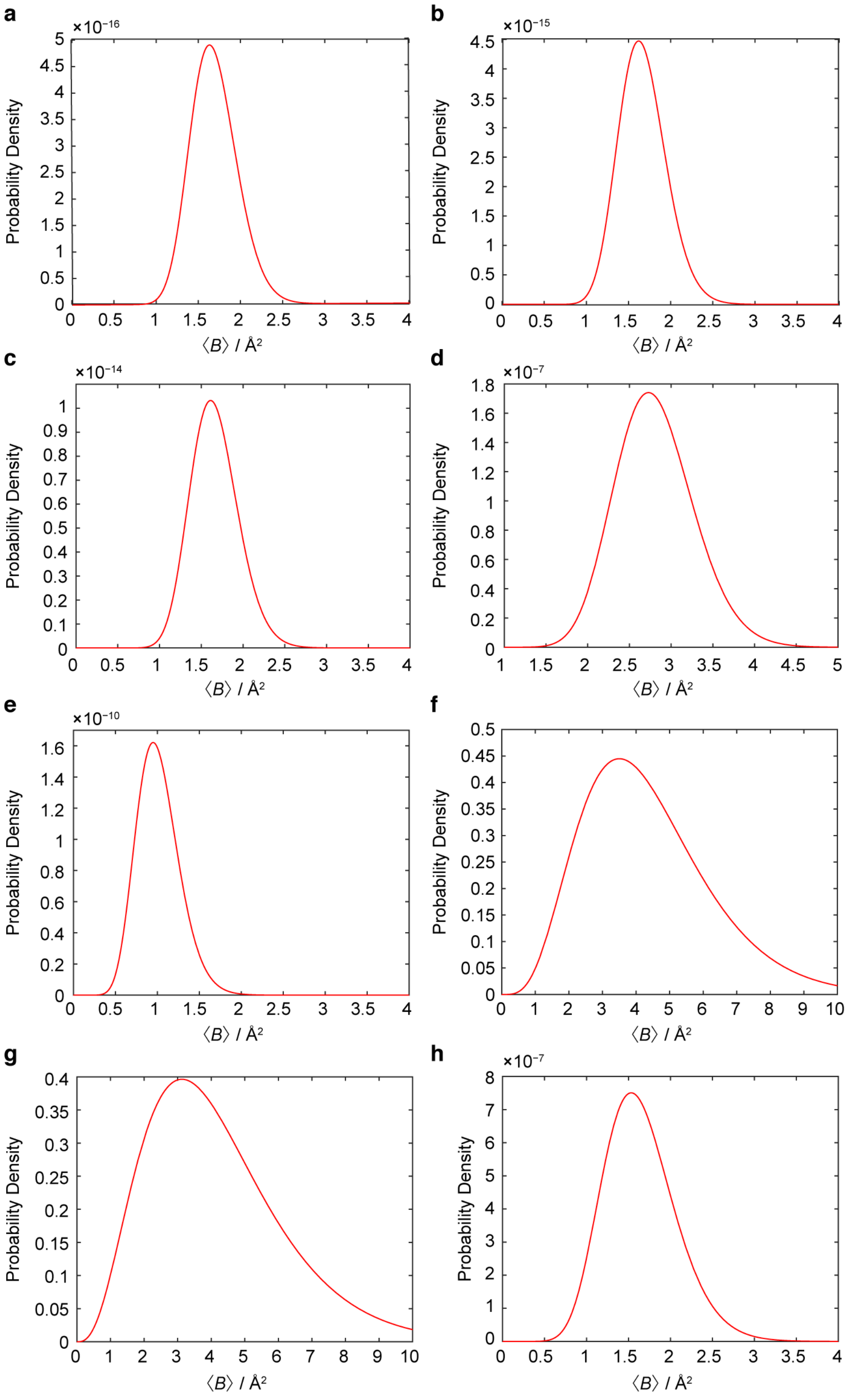

Supplementary Fig. 5| Calculated posterior distributions of $\langle B \rangle$. Probability distribution of $\langle B \rangle$ given by the subject (**a**), the excluded reference (**b**), the reference (**c**), the first masked (**d**), and the second masked (**e**) data set of **1**, as well as the data set of **2A** (**f**), **2B** (**g**), and **3** (**h**).

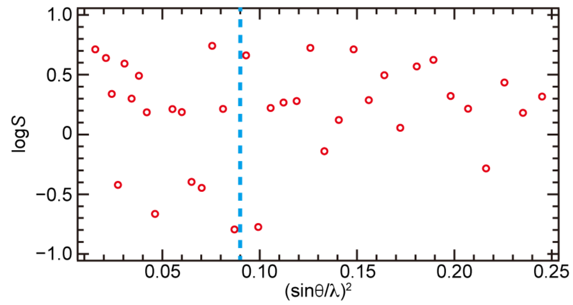

Supplementary Fig. 6| Plot of the logarithms of *S* toward the square of the resolution (Wilson plot) using the preliminary data set of 3. The chosen threshold resolution ($\sin\theta/\lambda$ = 0.300 Å^{-1}) is shown by the blue dashed line.
